# Isochores and Heat Capacity of Liquid Water in Terms of the Ion–Molecular Model

**DOI:** 10.3390/ijms24065630

**Published:** 2023-03-15

**Authors:** Alexander A. Volkov, Sergey V. Chuchupal

**Affiliations:** Prokhorov General Physics Institute of the Russian Academy of Sciences, Moscow 119991, Russia

**Keywords:** liquid water, modeling, heat capacity, isochores, equation of state

## Abstract

Thermodynamics of liquid water in terms of a non-standard approach—the ion–molecular model—is considered. Water is represented as a dense gas of neutral H_2_O molecules and single charged H_3_O^+^ and OH^−^ ions. The molecules and ions perform thermal collisional motion and interconvert due to ion exchange. The energy-rich process—vibrations of an ion in a hydration shell of molecular dipoles—well known to spectroscopists with its dielectric response at 180 cm^−1^ (5 THz), is suggested to be key for water dynamics. Taking into account this ion–molecular oscillator, we compose an equation of state of liquid water to obtain analytical expressions for the isochores and heat capacity.

## 1. Introduction

The molecular structure of liquid water, despite decades of scientific effort, remains debatable. The current view is contradictory. On the one hand, in educational problems, water is considered on a par with other liquids as an ensemble of free molecules [1]; on the other hand, in the specialized literature, its properties are associated with a special property—the presence of a network of hydrogen bonds. According to the second view, all the richness of water properties is due to the dynamic processes in the structure of hydrogen bonds—directed intermolecular interactions, their constant breaks and formations [2,3,4]. The study of the hydrogen-bond (HB) network is considered to be of strategical importance: “What is the structure and dynamics of the network of hydrogen bonds in water, which is responsible for its unique properties? This issue has been discussed for over 100 years and has not yet been resolved” [5]. HB-based data interpretation constitutes the research mainstream.

In a series of articles, we developed a non-standard approach—the ion–molecular (IM) model of liquid water—in which the HB concept is not used [6,7,8,9]. Instead, we suggest that the H_2_O molecules are connected by Coulomb fields of short-lived H_3_O^+^ and OH^−^ ions. The idea of the ion–molecular cohesion of water is outside the mainstream, but is physically clear and permits reliable comparison with experimental data. So far, we have tested it in terms of electrodynamic and transport properties of liquid water [9]. In this work, we analyze the IM model in the thermodynamic aspect—we compose the equation of state to obtain analytical expressions for the isochores of liquid water and heat capacity.

Despite the vastness of the accumulated experimental and theoretical material, water, surprisingly, is not a model object. A myriad of particular water-related problems are considered in reviews and textbooks, but the typical results are scattered [10], conclusions are not far-reaching, and they do not provide a holistic view of the microscopic liquid water design [11]. As a result, a vague idea of an extremely complex structure of liquid water is accepted. The popular belief is that “liquid water is regarded as consisting of both of bound ordered regions of a regular lattice and regions, in which the water molecules exhibit a hydrogen-bonded-type random array, which is interwoven by monomeric water, random holes, lattice vacancies, and cages” [11]. Due to the ambiguity and randomness of the above structure, its uniform and consistent analysis appears to be a daunting challenge: “It is well known that it is not possible to fit the overall water properties with a single set of parameters. Otherwise, there would be no explanation for the vast number of water models proposed in the literature” [12].

Presently, there is another water-related picture, more unambiguous and regularized—the electrodynamic response of water in the form of broadband dielectric spectra (frequency dependencies of the dielectric constant and/or conductivity). An experimental method for obtaining such data is dielectric spectroscopy (DS) [13,14,15,16]. This method reveals the energy behavior of matter particles in an unprecedentedly wide frequency range—10^−5^–10^15^ Hz, including audio and radio frequencies, microwaves, and IR range. Typically, frequency panoramas are filled with many spectral features suitable for reliable modeling [17].

For water, DS data has been the basis for speculations for many years [18,19,20,21,22,23,24,25,26]. Gradually, interpretations of dielectric spectra have become more general, but the underling HB concept has not changed. On this basis, inconsistencies accumulate, and the spectral pattern remains ”puzzling” [25]. In turn, we considered the DS data to provide a real chance to start with a clean slate. Somewhat surprisingly, the picture we constructed in this way turned out to be radically different from the accepted one.

## 2. Results and Discussion

### 2.1. The IM Model of Liquid Water

**Figure 1:**
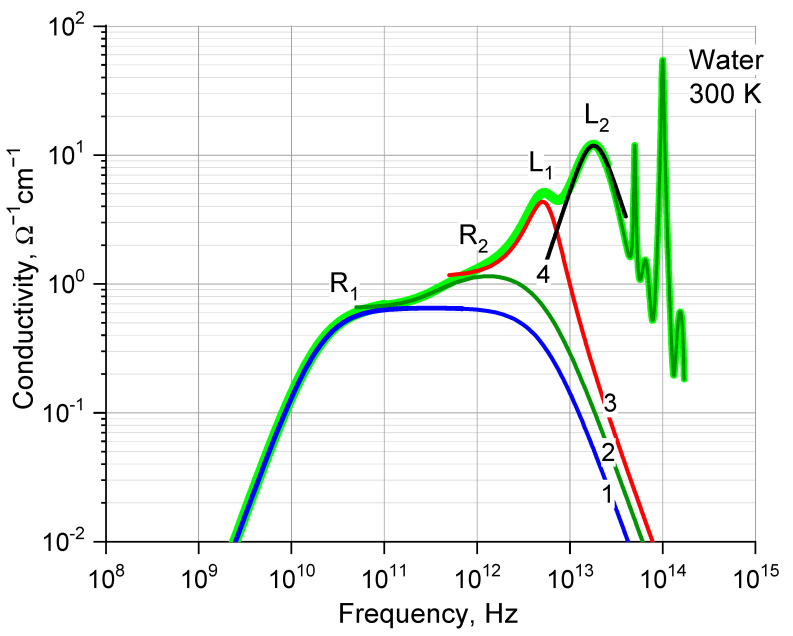
The model spectral pyramid of the dielectric response of liquid water (see also Figure 10 in Ref. [27]) superimposed on reference data obtained from Ref. [28]. Numbers 1–4 notate separate spectral components. Letters are the common notations of the spectral bands (R_1_ is famous Debye relaxation).

The outline of our reasoning for the IM model is as follows. Figure 1 shows the dielectric response of water in terms of conductivity. In Ref. [27], we described it analytically by a set of strongly damped oscillators, arranged in a pyramid and interconnected. We interpreted the IR active pyramid as a fingerprint of the oscillatory motion of nested charged molecular structures.

**Figure 2:**
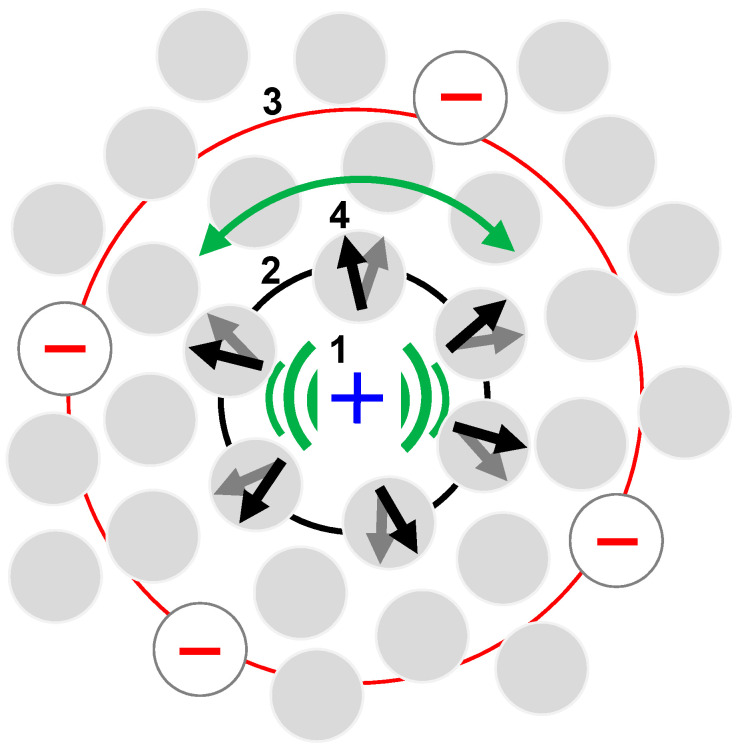
The 5-THz molecular oscillator in liquid water. 1—the vibrating H_3_O^+^ ion (see also Figure 12 in Ref. [27]); 2—the hydration shell; 3—the ion cloud; 4—librating H_2_O dipoles (black/grey arrows); shown are the ion vibrations (green rattling) and arrows swinging (green double arrow). Shadowed circles are H_2_O molecules; open circles are ions OH^−^; red and blue signs “+” and “− “ show the ion charges.

In the application to the liquid water structure (Figure 2), the motion is a domino-like chain of molecular processes—the oscillation of oxygen (O) inside the hydration shell (L_1_), the deformation oscillation of the hydration shell (R_2_) and the deformation oscillation of the ion cloud (R_1_). The left slopes of the oscillators in Figure 1 add up to the solid absorption spectrum of water, and the right slopes demonstrate the motion chain genesis—the intermolecular collisions in the region of 18 THz. The O–O collisions are accompanied by the supramolecular O–O proton transfer, which is the initial, triggering mechanism of the general coherent molecules motion. Peak L_2_ reflects the fastest reaction of the environment to the O–O transfer—concerted librations of molecular dipoles.

From the dynamics point of view, the L_1_ movement is the main energy container while R_2_ and R_1_ are the space-time waves diverging from it. The two latter energies are of a smaller order of magnitude and we will not take them into consideration further. We consider the final IM model of liquid water to be a medium consisting of neutral dipole H_2_O molecules plus singly charged H_3_O^+^ and OH^−^ ions. The ions perform oscillatory collisional motion inside the cage of center-symmetrically arranged polarized H_2_O molecules. The molecular swarms thus formed make bipolar Brownian motion. Due to the fast proton exchange, the molecular pattern changes rapidly but strictly systematically to permit characterization of a system by averaged well-defined parameters.

### 2.2. The Basic Postulate

Our task is to evaluate the thermal energetics of the molecular structure shown in Figure 2. We postulate the dense packing condition

(1) $$Nl^{3}=\sqrt{2},$$

where *N* is the concentration (number density) of H_2_O molecules and *l* is the intermolecular O–O distance. Thermodynamic parameters—energy *E*, pressure *P* and heat capacity *C*—are expressed through *N*, *l*, and volume *V* by the interconnected relations:

(2) $$NV=\frac{M}{m},\;V=\frac{M}{\sqrt{2}m}l^{3},\;n=\frac{N_{i}}{N},$$

where *N_i_* is the concentration of ions H_3_O^+^ and OH^−^; *M* and *m* are masses of a sample and of a single water molecule. The quantities *M* = 10^3^ kg (*N* = 3.3∙10^28^ m^−3^ for *V* = 1 m^3^), *m* = 3.1∙10^−26^ kg, *q* = 1.6∙10^−19^ C—the elementary charge, *p* = 6.14∙10^−30^ C·m—the dipole moment of the H_2_O molecule, and ε_0_ = 8.85∙10^−12^ F∙m^−1^—the dielectric constant of vacuum were used in calculations. Note that our working *M* = 10^3^ kg is equivalent to 55.5 kmol of particles.

To build the vibration energy of the molecular structure shown in Figure 2, we first refer to our important finding reported in Ref. [9]. We discovered that the cohesion energy of molecules in liquid water can be expressed by a simple formula for the electrostatic ion–dipole interaction [29]:

(3) $$E_{qp}=\frac{qp}{4\pi \varepsilon_{0}l^{2}}.$$

**Figure 3:**
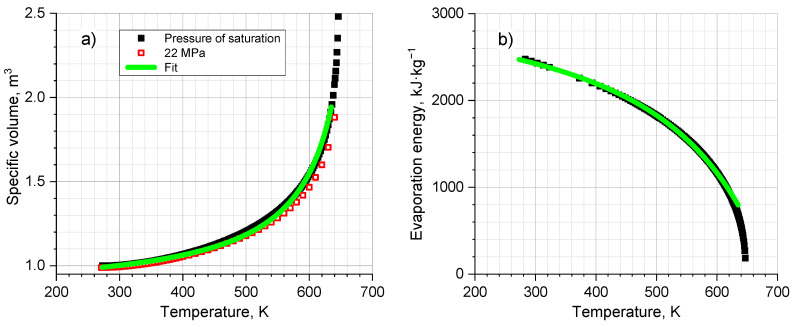
(**a**) Water specific volume *V* (1 m^3^ at 300 K) and (**b**) evaporation energy *E_VAP_* vs. temperature. Black points are reference data obtained from Ref. [30]. Solid lines are the fitted dependence *V*(*T*) in (**a**) panel and the same *V*(*T*) recalculated into the dependence *E_VAP_*(*T*) in (**b**) panel by the electrostatic formula (4).

The energy in the form of Equation (3) multiplied by the concentration *N* taking into account Equation (2) becomes

(4) $$E_{VAP}=\frac{B}{2^{4/3}}N^{5/3}=\frac{B}{2^{4/3}}\frac{M^{5/3}}{m^{5/3}V^{5/3}}=2.42\cdot 10^{9}V^{-5/3},$$

where $B=qp/(2\pi \varepsilon_{0})$ for compactness. *E_VAP_* is the energy of decomposition of water into separate molecules, the energy of evaporation. Equation (4) is retained over a wide temperature range, 273–630 K. What is important and convenient for us, due to the incompressibility of water, is the fact that volume *V* practically does not depend on pressure (see Figure 3a). This makes it possible to use a simple fitting temperature dependence $V(T)=73\cdot {\left(700-T\right)}^{-1}+0.82$ m^3^ (hereafter, temperature expressed in Kelvin).

The fitting work of the formula and calculations on Equation (4) are illustrated in Figure 3. Notably, Figure 3a,b are converted into each other according to the school formula without a single fitting intervention. It seems that we have the first, if not the only, non-trivial, quantitative expression for the internal energy of a molecular system, notably such important as water. We use simplified fitting formulas and the tactics of minimal fitting intervention throughout our present work.

An exhaustive analytical and physically clear description of the energy *E_VAP_* makes it possible to confidently rely on the virial theorem. This theorem states that for a system of interacting particles in a state of dynamic equilibrium, in the case when all forces acting on the particles are internal with respect to the system and are inversely proportional to the square of the distance (just our Equation (3)), the average kinetic energy of the particles is equal to half the average value of the potential energy of the system of interacting particles, taken with the opposite sign [31].

Based on the virial theorem, we can use the principle of equipartition of energy over degrees of freedom, namely we can consider the energy (4) to be equally divided between the kinetic energy of free molecules and the kinetic energy stored in oscillators (a kind of potential energy). The second, oscillatory half, in turn, can be considered distributed between the rapidly interconverted kinetic and potential energies of an oscillator. We continue the topic of equipartition of energy in Section 2.6.

### 2.3. The 5-THz Molecular Oscillator

Equation (4) produces the binding energy per molecule 7.2∙10^−20^ J. The new finding of the present work is that the molecular oscillator shown in Figure 2 has the energy of the same order of magnitude. In fact, in the harmonic approximation

(5) $$E_{Z}=\frac{3}{2}m\omega_{Z}^{2}\lambda^{2},$$

*ω_Z_* is the eigen circular frequency, *λ*—the amplitude and, as we set, $\kappa =m\omega_{Z}^{2}$—the elasticity coefficient. Index *Z* means Zelsmann’s oscillator studied in detail in Ref. [32] by the method of dielectric spectroscopy. The temperature dependence of the frequency was established. We employed it for our research in the form of the fitted curve $\nu_{Z}=(8.1-0.0095\cdot T)\cdot 10^{12}$ Hz to calculate $\kappa =102-4\cdot T^{1/2}$ kg∙s^−2^ (for reference, *κ* = 33 N∙m^−1^ at room temperature).

**Figure 4:**
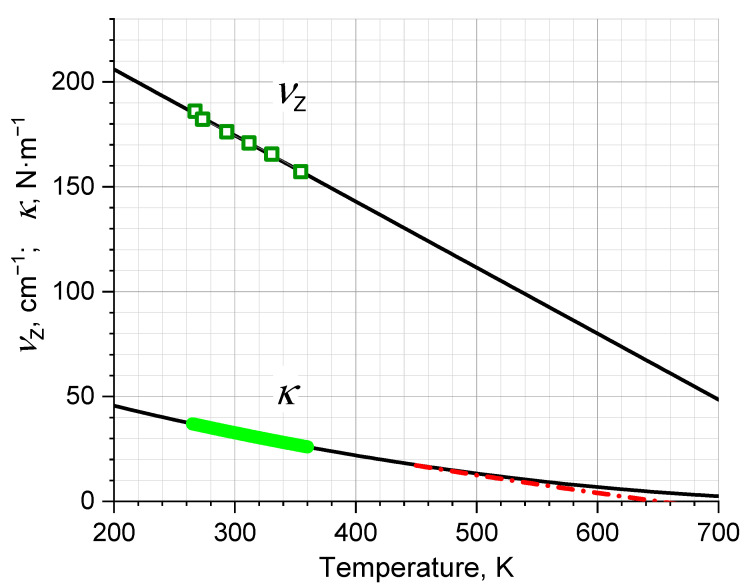
The 5–THz oscillator eigen frequency *ν_Z_* (**top**) and elasticity constant *κ* (**bottom**) vs. temperature. Squares are the data obtained from [32], the thick segment shows the operating range of the experiment, the lines show extrapolations beyond the range. The dashed line is forced extrapolation to the critical point 647 K.

The calculation stages are shown in Figure 4. First, we suggested that κ depends on *T* in so far as depends on *V*, more precisely, on $l\sim V^{1/3}$. Then, we converted the temperature dependence *ν_Z_*(*T*) into the dependence *ν_Z_*(*V*), to calculate the dependence *κ*(*V*) and then *κ*(*T*) with further forced extrapolation to the critical point 647 K.

We set the oscillation amplitude λ to be the mean free path of molecules and ions. According to gas-kinetic consideration $\lambda =1/\left(\sqrt{2}N\sigma \right)$ [33], where $\sigma =\pi d^{2}$ is the scattering cross section and *d* is the diameter of a molecule, 2.8 Å is for water [34]. For the full volume, taking into account Equation (2), we obtain

(6) $$E_{OSC}=N_{i}E_{Z}=\frac{3}{4\sigma^{2}}n\kappa \frac{1}{N}=\frac{3}{4\sigma^{2}}n\kappa \frac{m}{M}V.$$

The oscillator under discussion obviously has a cohesive property: it is formed by molecular dipoles which are pulled together by a strong inhomogeneous central Coulomb field (ion–dipole interaction).

### 2.4. Thermodynamic Outline

Now, let us introduce the oscillator energy into thermodynamics. We proceed from what is very much present in textbooks and the specialized literature [1,35,36,37,38,39,40]. We consider the internal energy of liquid to be the sum of kinetic energies of individual particles and the interaction energy between particles. In an isochoric process, all heat contributes to increasing the internal energy U.

The fundamentally important ratio is $dU={\left(\partial U/\partial T\right)}_{V}dT+{\left(\partial U/\partial V\right)}_{T}dV$ or $dU=C_{V}dT+P_{i}dV$, where $C_{V}={\left(\partial U/\partial T\right)}_{V}$ is the heat capacity and $P_{i}=-{\left(\partial U_{i}/\partial V\right)}_{T}$ is the internal pressure. The latter, *P_i_,* is a measure of cohesion forces in a substance determined by energy interaction $U_{i}$ between molecules. The mentioned parameters are linked by the equation of state. They are varied. Integration provides $U=\int C_{V}(T)dT+U_{i}$ for energy and $P+P_{i}=T{\left(\partial P/\partial T\right)}_{V}$ for pressure. Zero *P_i_* reduces the equation of state to that for an ideal gas, PV/T=const.

A famous van der Waals equation, the simplest and most physically clear, is $P=Nk_{B}T\left(V-b\right)-P_{i}$, where $P_{i}=a/V^{2}$ and the corresponding energy $U_{i}=-a/V$. The coefficient *a* models the attractive interactions between molecules while the coefficient *b* accounts for the total volume of the molecules. The parameter *a* does not depend on temperature and van der Waals’s heat capacity does not depend on volume.

### 2.5. Isochores and Heat Capacity

In view of the above, *P_i_* seems to us to be the value which can be substituted by $P_{OSC}=-{\left(\partial U_{OSC}/\partial V\right)}_{T}$ to incorporate the oscillator into thermodynamics. In this case, both *C_V_* and *P_i_* depend on both *V* and *T*, but we consider the relationship *V*(*T*) to be known (see Section 2.2). Then, our equation of state, by analogy with van der Waals equation and taking into account Equation (6), can be written as

(7) $$P=\frac{3}{2}Nk_{B}T-P_{i}\Rightarrow \frac{3}{2}\frac{1}{V(T)-b}\frac{Μ}{m}k_{B}T-P_{OSC}\left(V,T\right).$$

**Figure 5:**
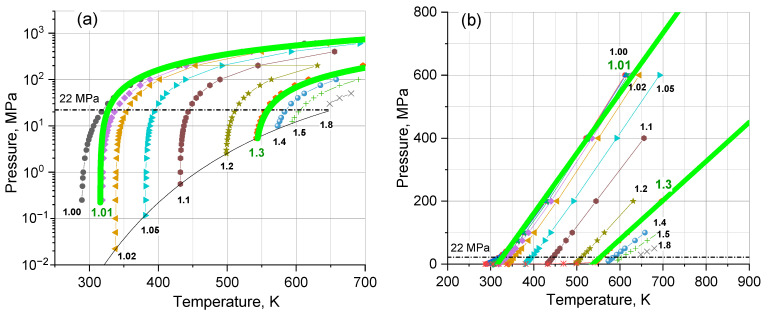
Isochores of liquid water in (**a**) log and (**b**) normal scales. The solid thick lines are the representative curves calculated according to Equation (7) for *V* = 1.01 and 1.3 m^3^ (*N* = 900 and 770 m^−3^). Reference points are taken from Ref. [30].

How does it relate to reality? We clarified this issue by calculating on Equation (7) the water isochores obtained from the database [30]. For *V*-values as parameters in the range of 1–5 (i.e., for temperatures of 300–700 K), we selected *b* and *P_OSC_*(*V*) to fit the experiment. We determined the predictions of Equation (7) to be satisfactory at a constant *b* = 0.68 ± 0.02 m^3^ (or 0.0123 m^3^·kmol^−1^) when changing *P_OSC_*(*V*) according to the law $P_{OSC}\left(V\right)=800/V^{1.4}$ MPa or, in terms of temperature, $P_{OSC}\left(T\right)=800/{\left[73\left(700-T\right)+0.82\right]}^{1.4}$ MPa. The quality of the fit is illustrated in Figure 5.

Integration of Equation (7) over *V* leads to the equation of state for energy:

(8) $$U=\int PdV=\frac{3}{2}\frac{M}{m}k_{B}T\left[\ln\left(V(T)-b\right)+c\right]+E_{OSC}\left(V,T\right),$$

where *c* is the constant of integration. This equation is a nodal one. By inverse differentiation with respect to *V*, we would obtain the pressure, but with differentiation with respect to *T* we obtain the heat capacity:

(9) $$C_{V}=\frac{3}{2}\frac{M}{m}k_{B}\frac{\partial \left\{T\cdot \left[\ln\left(V(T)-b\right)+c\right]\right\}}{\partial T}+P_{OSC}{\left(\frac{\partial V}{\partial T}\right)}_{U}.$$

**Figure 6:**
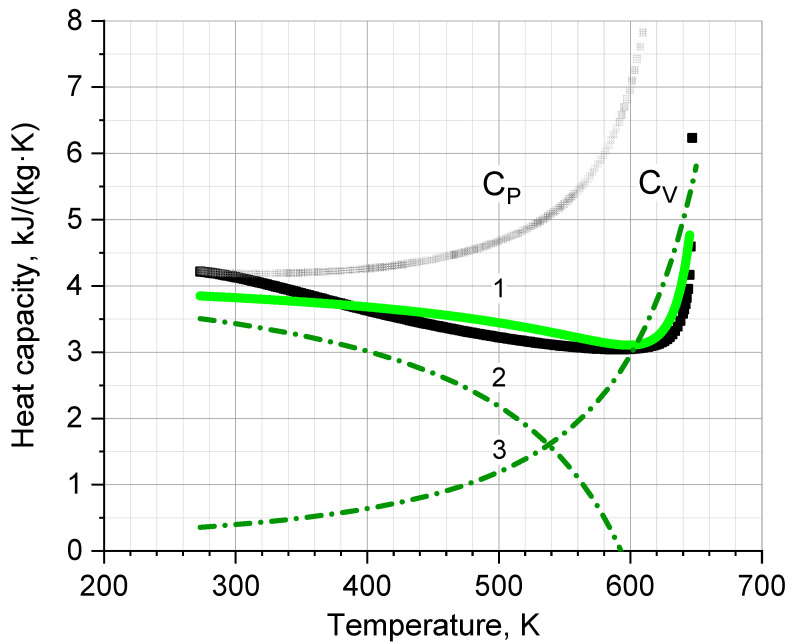
Heat capacity *C_V_* of liquid water vs. temperature. The solid line 1 is the fitting curve of Equation (9) to reference points of *C_V_* obtained from Ref. [30] (square points). Dashed lines 2 and 3 are the components of the *C_V_*(*T*) curve 1. *C_P_* is shown for completeness.

The only fitting parameter c=−4±0.5 turns out to be sufficient. It provides, together with the previously determined *b* = 0.68 m^3^ and the dependence *P_OSC_*(*T*), good agreement with reference *C_V_*(*T*), as shown in Figure 6.

To reiterate, we used the parameters of the electrodynamic 5-THz oscillator to postulate the thermodynamic van der Waals-like equation of state and, with its help, consistently evaluate the isochoric pattern and the heat capacity of liquid water. The calculated data fitted satisfactorily with reference data.

### 2.6. The Ion Concentration

**Figure 7:**
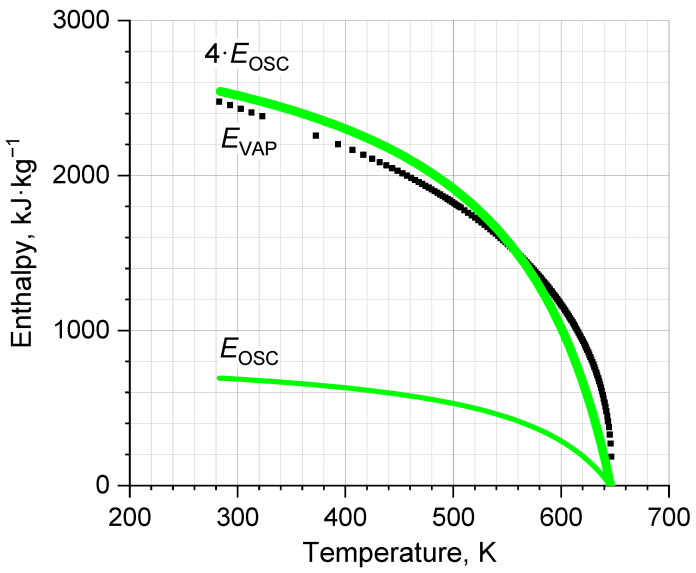
Comparison of the potential energy of an oscillator *E_OSC_* and the energy of decomposition (evaporation) of liquid water *E_VAP_* [30] to demonstrate the prediction of the equipartition principle: $E_{OSC}=1/4E_{VAP}$.

The grain of our consideration is the quantity *nκ*, which enters into the energy *E_OSC_* (6). It contains information about the concentration of ions $N_{i}=nN$ which are fundamentally inherent in our IM model. To evaluate *n*, we considered *E_OSC_* to be the maximal potential energy of an oscillator and, at same time, of the part of the total potential energy *E_VAP_*. Then, the energy *E_OSC_*(*T*) as an integral of the dependence $P_{OSC}=-{\left(\partial U_{OSC}/\partial V\right)}_{T}$ can be compared with *E_VAP_*(*T*). Figure 7 clearly shows the dependence *E_OSC_*(*T*) to be a quarter of *E_VAP_*(*T*).

The result is consistent with that dictated by the equipartition principle, discussed earlier in Section 2.2. Namely, according to the equipartition principle, the total energy is distributed between the molecular and oscillatory systems, while the latter divides its energy in twain between kinetic and potential energies. Then, we postulate $E_{OSC}=1/4E_{VAP}$ and, taking into account Equations (4) and (6), we obtain the formula

(10) $$n\kappa =\frac{B\sigma^{2}}{3\cdot 2^{4/3}}N^{8/3}=\frac{B\sigma^{2}}{3\cdot 2^{4/3}}{\left(\frac{M}{m}\right)}^{8/3}V^{-8/3}.$$

**Figure 8:**
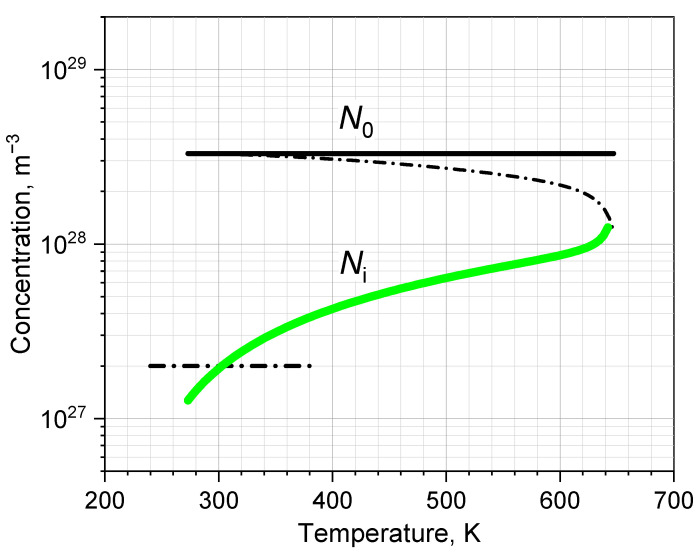
The ion concentration *N_i_* in liquid water. The mark *N_i_* = 2·10^27^ m^−3^ shows a level of previous *N_i_* of the IM model [9]. The total particle concentration *N*_0_ is shown for comparison (solid—isochoric, dashed—along the critical water–vapor curve).

Given the above settled dependence *κ*(*T*) (see Section 2.3), we calculate *n*(*T*) and then—$N_{i}=nN$. The result is shown in Figure 8. Strikingly, the ion concentration is quite a bit, about an order of magnitude, inferior to the concentration $N_{0}-N_{i}$ of neutral H_2_O molecules.

### 2.7. The Ion Concentration Issue

In our IM model, thermodynamics is determined by the ion concentration *N_i_* as well as the specific elasticity *κN_i_/N* by its value and temperature behaviour on the *PVT* surface. The parameters *n* and *κ* are new ones, which do not exist in common HB models.

Now, we proceed on the basis that the IM model successfully describes the reference data in Figure 5 and Figure 6 and predicts the ion concentration shown in Figure 8. By this we assume the adequacy of the model, which is necessary for the performed fittings to be meaningful. As can be seen, the IM model produces a very high concentration of ions *N_i_* in water (higher than the level 2∙10^27^ m^−3^ in Figure 8), the one that we invariably obtained in our previous works [6,7,8,9]. Thus, the success of the IM model once again appears to be due to the high concentration of ions in liquid water *N_i_*, which performs a cohesive function and determines the thermodynamic properties of water. Notably, this high concentration *N_i_* is the constant basic objection to the IM model. A typical objection is “the model, however, requires a hydronium ion concentration by seven orders of magnitude higher than the actual concentration [H_3_O^+^] = 10^–7^ mol/dm^3^ and appears thus implausible” [41]. Here, let us pay attention to the fact that the “actual concentration” *N_i_* = 10^–7^ mol/L was adopted conventionally based on the results of many disparate measurements (conductometric and chemical), which were interpreted using a whole set of assumptions. The main assumption was that there are few H_3_O^+^ and OH^−^ ions in liquid water and they do not interact with each other [42]. Then, under conditions of high ion mobility originating from chemical experiments, the low static conductivity of water is naturally interpreted as evidence of low ion concentration.

In our IM model, the molecular dynamic is different—particles interact strongly and complexly; on a picosecond time scale, they are mutually interconverted. Charged particles—oxygen atoms with access proton (or hole)—mobilize swarms of dipole H_2_O molecules around themselves (marked by arrows in Figure 2). Additionally, there is an electrophoretic effect due to the interaction of an ion with the surrounding counterions (interaction of “+” with a few “−”). During thermal collisions, the charge transfer occurs (the O–O proton/hole transit) resulting in the rearrangement of the environment in a new equilibrium state. In Figure 2, this is displayed as a charge “+” jump to the neighboring molecule (with an arrow) and the subsequent relaxation of the environment (molecular swarm) until the pattern is completely restored in a new equilibrium state (having been shifted by one random intermolecular step). Random O–O proton jumps and rearrangements of the swarms constitute the steps of the chaotic swarm wandering, i.e., the motion of an oscillator with the Brownian mobile centrum.

We note, although our Equation (7) is structurally close to the van der Waals equation, it is built according to a completely different arguments, suggesting a high ionic concentration. Interestingly, the van der Waals equation also leaves room for such an idea: “In a liquid, van der Waals’ “*a*/*V*^2^” takes charge: the molecules are tied by attractive forces in a semi-organized, mobile, crowd; there must be some local order among neighbors, but not the permanent seating of a solid crystal” [43].

## 3. Materials and Methods

### 3.1. General Strategy

In this work, we searched for the possibility of describing thermodynamic properties of liquid water by a simple and physically clear methods. We used as an experimental data to be predicted the data taken from databases [28,30]. Simple basic analytical relations of classical electro- and thermodynamics were used for the modeling [13,37].

Commonly, models of new materials are expected to predict new properties. In the case of water, the situation is special—water’s properties have already been studied thoroughly and they are known in great detail. This reduces the chance of a new discovery but provides a basis for exhaustive validation of the model. Following this tactic, we seek agreement of model calculations with the rich body of reference data accessible in the literature. The space for fitting is huge. The situation is dynamic; there are many branching points in logical constructions. The success of the model is in the maximum grasp of everything known. At the first stage, we chose isochores and the heat capacity of liquid water as the most convenient for fitting.

### 3.2. Resolution of the 180 cm^−1^ (5-THz) Peak Issue

The complex molecular motion which is invented by us to agree numerous microscopic properties of water with the electrodynamic response of water shown in Figure 1 (see. Ref. [27] on this issue) demonstrates at the same time, a rather simple and physically clear fact, namely the presence of a local energy-saturated oscillator in water which allows simple parametrization. Thus, the known spectral peak at 180 cm^−1^ (5 THz) assumes a worthy place in the problem of its application to thermodynamics. 

It should be noted that 180 cm^−1^ peak is special in spectroscopy (infrared, Raman, nuclear scattering). For many years, it presented an insoluble problem for the molecular interpretation [44]. Many arguments have been proposed in favor of different models, mainly focused around that, we quote, the 180 cm^−1^ peak is related to hindered longitudinal translations of water molecules in the HB network with a distinct collective character [45], that it arises from the molecular translation modes [46], that it is due to the stretching vibrations of nearly linear hydrogen bonds, or due to cage vibrations or more exotic hydrogen bond network relaxations [23], that it is due to the presence of water with a tetrahedral hydrogen bond conformation [47], that it is due to a coupling between the translational degrees of freedom of the water molecule and the electronic cloud [48].

Remarkably, generating the peak at approximately 180 cm^−1^ in the low-frequency infrared spectrum for liquid water “is an intrinsic difficulty in modeling condensed-phase water with conventional rigid non-polarizable water models” [49]. However, “even polarizable models fail in reproducing this feature” [48].

In terms of the IM model, the problem of the 180 cm^−1^ peak seems to be rather clear. Against a background of endless discussions, the main feature of the 5-THz peak for us is that it shows no H–D isotope effect [32]. Just a large mass of a charged species and the specific temperature dependence of the frequency (Figure 4) distinguishes the spectrally modest 5-THz peak (L_1_, Figure 1) as an energy-saturated oscillator. We can assume that all water molecules vibrate within their environment, and those that are currently marked by the presence of an excess or missing proton, that is, are charged, respond in the IR spectra. An oxygen atom with an odd number of protons is IR active. The 5-THz oscillation is presented as a local resonant one, similar to those known in solid state physics (excitons, polarons) [50]. The localization time (ion life), according to our data, is on the scale of picoseconds [27].

### 3.3. Striving for Simplicity and Clarity

Our work is conducted in line with a long-overdue need—to seek “how water molecules move in between each other and not always search for the determining structure” [51]. The IM model just assumes the sufficient free movement of molecules with translational kinetic energy, which contributes to the acceptance of heat and its retention. According to Feynman, “the problems of molecular motion are so complicated that even an elementary understanding, although inaccurate and incomplete, is worthwhile having. The real successes come to those who start from a physical point of view” [52].

With this approach, we attempted to consider the thermodynamics of liquid water within the framework of an ion–molecular model inspired by the panoramic dielectric spectra of water [27]. A specific effect taken from dielectric spectroscopy is introduced into the model—an energy-enriched oscillator at 5 THz. It is assumed to be a vibration of a singly charged H_3_O^+^ or OH^−^ ion in a cage of dipole H_2_O molecules swarming around it.

The main result of the present work is a satisfactory description of a large body of experimental data with the help of few fitting parameters. This is important because the IM model is fundamentally different from all that are presented in the literature. While the latter derive from the radial distribution function, static in essence, the IM model originates from dynamic data on the electrodynamic response of liquid water. Initially, at a very early stage, since the time of Röntgen, the radial distribution functions of water showed the molecular water structure similar to that of polymorphous SiO_2_ [53]. Further spectral measurements, up to presnt, are interpreted in the already accepted paradigm.

The modern water modeling develops predominantly through computer simulation techniques with increasing attention to fine-tuning and quantum mechanical detail [12,44,48]. We are more inclined towards the tactics of simplified analytical models, in which “the chain of logic from the model premises to conclusions is so much more transparent”, we share the opinion that “there is a need for computationally cheaper models of water that can retain the relevant physics” [54].

## 4. Conclusions 

We used the spectroscopic data as dynamic input to turn to the fundamental principles and to find an alternative view on water structure. Namely, we addressed the similarity of the electrodynamics of liquid water with the electrodynamics of electrolytes to develop a new ion–molecular concept. Although the model is microscopically complex, it is physically quite clear. This made it possible to use averaged values to form proper postulates and connect the 5 THz electrodynamic feature with general thermodynamics. The good agreement between the calculated isochores of water and heat capacity is a valuable argument in favor of the IM model and stimulus for its further development.

## Data Availability

Data can be made available by the authors upon reasonable request.

## Abbreviations

The following abbreviations are used in this manuscript:

HB	hydrogen bond
IM	Ion–molecular
DS	dielectric spectroscopy
IR	infrared
